# Coverage and impact of influenza vaccination among children in Minhang District, China, 2013–2020

**DOI:** 10.3389/fpubh.2023.1193839

**Published:** 2023-08-30

**Authors:** Zhaowen Zhang, Liming Shi, Nian Liu, Biyun Jia, Kewen Mei, Liping Zhang, XuanZhao Zhang, Yihan Lu, Jia Lu, Ye Yao

**Affiliations:** ^1^Minhang Center for Disease Control and Prevention, Shanghai, China; ^2^School of Public Health, Fudan University, Shanghai, China; ^3^Key Laboratory of Public Health Safety of Ministry of Education, Fudan University, Shanghai, China

**Keywords:** influenza, vaccination, children, averted cases, burden, China

## Abstract

**Background:**

Young children have a great disease burden and are particularly vulnerable to influenza. This study aimed to assess the direct effect of influenza vaccination among children and to evaluate the indirect benefit of immunizing children.

**Methods:**

The influenza vaccination records for all children born during 2013–2019 in Minhang District and surveillance data for reported influenza cases were obtained from the Minhang CDC. 17,905 children were recorded in the vaccination system and included in this study. Descriptive epidemiology methods were used for data analysis, including an ecological approach to estimate the number of influenza cases averted by vaccination and linear regression to estimate the reduction in influenza cases in the general population per thousand additional childhood vaccination doses.

**Results:**

During the study period, the annual vaccination coverage rate ranged from 10.40% in 2013–2014 to 27.62% in 2015–2016. The estimated number of influenza cases averted by vaccination ranged from a low of 0.28 (range: 0.23–0.34) during 2013–2014 (PF: 6.15%, range: 5.11–7.38%) to a high of 15.34 (range: 12.38–18.51) during 2017–2018 (PF: 16.54%, range: 13.79–19.30%). When increasing vaccination coverage rate by 10% in each town/street, a ratio of 7.27–10.69% cases could be further averted on the basis of observed cases. In four selected periods, the number of influenza cases in the general population was most significantly correlated with the cumulative childhood vaccination doses in the prior 2–5 months, and the reduction in influenza cases ranged from 0.73 to 3.18 cases per thousand additional childhood vaccination doses.

**Conclusion:**

Influenza vaccination among children is estimated to have direct effects in terms of averted cases and might provide an underlying indirect benefit to the general population. Vaccination coverage in high-coverage areas should be further expanded to avert more influenza cases.

## 1. Introduction

Seasonal influenza is an infectious respiratory disease caused by influenza viruses and poses substantial morbidity and mortality annually (1). Young children have a great disease burden and are particularly vulnerable to influenza. It was estimated that 109.5 million influenza virus infections occurred worldwide in 2018 among children under 5 years of age (2). The highest influenza notification rates in Australia were observed among children aged 0–4 years (111 cases per 100,000 population compared with a total rate of 60 cases per 100,000 population) (3). The attack rate was highest among children aged 0–4 years (31.9%) for the 2015–2016 season in Beijing, China (4). Children have been identified as the main spreaders in influenza transmission. It was estimated that 40–48% of the secondary cases exposed to a child sick with influenza in the household are attributable to transmission from the child (5). In influenza B outbreaks, children aged 0–4 years had the highest estimated relative risk (6). Thus, relieving the disease burden among children will decrease the opportunity for influenza transmission to others.

Influenza vaccination is considered the most effective means of preventing influenza and can significantly reduce the risk of influenza and serious complications among vaccinated people (7, 8). An estimated 4.4 million illnesses and 58,000 hospitalizations were prevented due to influenza vaccination during the US 2018–2019 influenza season (9). Vaccinating children can protect them directly and is presumed to interrupt influenza transmission in the general population, which indirectly protects susceptible contacts (10). A cluster randomized controlled trial revealed that immunizing children aged 36 months to 15 years with inactivated influenza vaccine produced a protective effectiveness of 61% against confirmed influenza illness among unimmunized residents of communities (11). The Chinese Center for Disease Prevention and Control (Chinese CDC) has recommended annual seasonal influenza vaccines to be administered to children aged 6–59 months (12). However, because influenza vaccines are not included in the National Immunization Program (NIP) in most areas of China, vaccination coverage among children is relatively low (13). Influenza vaccination coverage among children aged under 5 years was estimated to range from 12 to 32% in China during 2009–2012 (14, 15).

Different models have been used to evaluate the impact of influenza vaccination on averted influenza-associated events (16–22). Averted events were defined as the difference between observed events and projected events in the absence of vaccination (23). Backer et al. developed a stochastic transmission model to estimate an average of 13% infections, 24% hospitalizations, and 35% deaths averted in the Netherlands (24). Zhang et al. used a dynamic transmission model to assess the impact of vaccinating school-going children in Beijing, China for the 2013–2016 seasons (19). Although these methods can take some factors into account, such as indirect effects, loss of immunity, and influenza activity variations between seasons, a series of parameters need to be estimated, and heavy computations are needed. Kostova et al. originally proposed a method to estimate the direct effect of influenza vaccination in terms of averted events in the US 2005–2011 seasons (21), and this method was then used and developed by some other researchers (18, 22, 25, 26). This method estimates the averted influenza cases and prevented fraction using three parameters: number of observed cases, vaccination coverage, and vaccine effectiveness. These measures to evaluate vaccine impact are easy to understand and interpret.

The objective of this study was to assess the direct effects of influenza vaccination among children by estimating the number of averted cases and prevented fractions, and to estimate the indirect effects by quantifying the relationship between cumulative vaccination doses among children and influenza cases in the general population.

## 2. Methods

### 2.1. Study design and population

In this study, based on multiyear vaccination records and surveillance data, we give observational evidences on direct and indirect effects of immunizing children. Children living in Minhang District, including permanent and nonpermanent residents, are registered on the National Immunization Program (NIP) system. The vaccination records of these children include demographic information such as age and sex and vaccination information such as vaccine type and vaccination time.

### 2.2. Data sources

#### 2.2.1. Vaccination data

We extracted the influenza vaccination records from July 2013 to June 2020 from the Minhang Center for Disease Prevention and Control (Minhang CDC) NIP system. One record was excluded due to a date of birth outside of the study period and 9 records were removed for their inaccurate vaccination dates. Finally, a total of 170,915 children who were born between January 2013 and December 2019 were enrolled in this study. Depending on the time pattern, we defined one vaccination year as being from July 1st to June 30th the next year. Thus, there were 7 vaccination years in this study from 2013–2014 to 2019–2020.

#### 2.2.2. Surveillance data

We obtained the surveillance data of confirmed influenza cases during 2016/01–2020/01 from the Minhang CDC Notifiable Infectious Diseases Reporting Information System, with missing data from April to November 2018. This dataset included age, sex, residential address, time of influenza onset, time of diagnosis, and flu types.

#### 2.2.3. Influenza incidence data

The monthly statistics of influenza in Shanghai during 2013–2018 were downloaded from the China CDC’s public health science data center (27). Influenza incidence by age group was used to estimate the number of observed influenza cases among children due to a lack of surveillance data before 2016 and missing data in 2018.

#### 2.2.4. Vaccination coverage

According to the China Technical Guidelines for Influenza Vaccines (12), children aged over 6 months can receive influenza vaccines. Yearly vaccination coverage (VC) was calculated using the vaccination data from the Minhang CDC by dividing the number of actually vaccinated children by the total population of children who met the vaccination criteria during the same period.

#### 2.2.5. Vaccine effectiveness

Influenza vaccine effectiveness (VE) varies widely in different seasons and influenza types and subtypes (28–30). Therefore, in this study, VE was assumed to be at a moderate level of 60%. We also performed a sensitivity analysis by adjusting the VE in an interval of +/− 10%. The results of the sensitivity analyses were presented as ranges of the estimated averted influenza cases to indicate uncertainties.

### 2.3. Averted influenza cases estimation method

To assess the impact of influenza vaccination on children, we estimated the number of averted influenza cases in two steps. First, the number of influenza cases among children during 2013–2018 was estimated by multiplying the monthly influenza incidence in each age group by the number of children who met the criteria for vaccination in that month. Yearly influenza cases were calculated by adding the monthly estimations together (details available in the supplementary file). The averted influenza cases were the difference between the expected influenza cases if there were no vaccinations given (N) and the observed burden with vaccination (n). The number of averted influenza cases (NAC) was then estimated using the following formula (22, 25):


$$NAC=N-n=\frac{n\cdot \left(VE\cdot VC\right)}{1-\left(VE\cdot VC\right)}$$

where n is the observed influenza cases, and VC and VE represent vaccination coverage and vaccine effectiveness, respectively.

The number of averted cases depends not only on VC and VE during that season but also on the influenza epidemic intensity, i.e., seasons with high epidemic intensity will result in a higher number of averted cases (21). Therefore, we estimated the prevented fraction (PF) as:


$$PF=\frac{NAC}{\left(NAC+n\right)}$$

a relative term measuring the impact of vaccination (22).

### 2.4. Statistical analysis

A chi-square test was performed to compare different features between vaccinated and unvaccinated groups. Pearson’s correlation analysis was performed between cumulative vaccination doses and influenza cases. Statistical analyses were performed in R language (version 4.1.3, R Core Team, Vienna, Austria).

### 2.5. Ethics statement

The study was reviewed and approved by the Institutional Review Board of the Minhang Center for Disease Control and Prevention. The number of the ethical letter regarding this study is EC-P-2020-010. Informed consent was waived for this study because it involved the use of surveillance data and no potentially identifiable human data were presented.

## 3. Results

During the study period, 170,915 children who resided in Minhang and were born between January 2013 and December 2019 were registered in the NIP system. Of these, 78,027 (45.65%) received at least one dose of influenza vaccine during the study period. The vaccination coverage rate among children with permanent residency was significantly higher than that among children with nonpermanent residency (47.45% vs. 43.85%, *p* < 0.001) (Supplementary Table S1). From 2013–2014 to 2015–2016, the annual influenza vaccination coverage rate increased nearly 3-fold from 10.40 to 27.62% (Table 1). The coverage rate rebounded to 27.57% after a slight fluctuation in 2016–2017. However, a sharp decline occurred in the following years, and vaccination coverage fell to 14.16% in 2018–2019 and 18.13% in 2019–2020. From the perspective of monthly trends, the peak vaccination period was from September to February the next year, with a minor period in August and March (Supplementary Figure S1). There were rare vaccinations in other months, except in 2019–2020. The vaccination coverage in each town was computed according to the children’s residential addresses on the records. During the study period, the lowest vaccination coverage rate was 6.11% on Pujin Street in 2016–2017, and the highest was 44.26% on Jiangchuan Street in 2017–2018 (Table 2). The mean coverage rates in each town over the study period ranged from 10.39 to 33.46%. The mean coverage rate in Minhang District during the study period was 21.93%, and the coverage rates of seven towns/streets were below the mean (Table 2). The coverage rates in Qibao Town, Meilong Town, Pujin Street, and Zhuanqiao Town were always below the average in each vaccination year, and the coverage rates in Meilong Town, Pujiang Town, and Pujin Street were 6 times below the lower quantile. In contrast, the coverage rates in Jiangchuan Street, Maqiao Town, and Xinhong Street were always higher than the yearly average.

**Table 1:** Yearly vaccination coverage rates in Minhang District from 2013–2014 to 2019–2020.

Year	Number of vaccinated children	Number of vaccination doses	The population of children in the same period	Vaccination coverage rates
2013–2014	2,563	4,670	24,652	10.40%
2014–2015	11,690	21,932	50,961	22.94%
2015–2016	19,984	37,156	72,356	27.62%
2016–2017	24,267	36,733	100,427	24.16%
2017–2018	34,757	54,208	126,054	27.57%
2018–2019	21,111	30,912	149,098	14.16%
2019–2020	30,991	40,930	170,915	18.13%

One vaccination year is defined as July 1st to June 30th in the following year. The study period was divided into 7 consecutive vaccination years from 2013–2014 to 2019–2020. Children aged 6 months and older could meet the criteria for receiving the influenza vaccine, and all children who met the criteria composed the population of children in the same period. Vaccination coverage was calculated by the number of vaccinated children divided by the population of children in the same period in each vaccination year.

**Table 2:** Annual and mean vaccination coverage rates in each town/street in Minhang District from 2013–2014 to 2019–2020.

Town/Street	2013–2014 (%)	2014–2015 (%)	2015–2016 (%)	2016–2017 (%)	2017–2018 (%)	2018–2019 (%)	2019–2020 (%)	Mean coverage rate
Huacao Town	10.53^†^	22.96	37.87	33.02	44.22	18.87	25.99	27.64
Qibao Town	6.29 ^††^	20.68	23.33	18.85	27.65	10.67	14.71	17.45
Hongqiao Town	14.18	22.35	30.17	27.39	27.40	18.94	22.78	23.32
Xinzhuang Town	9.33	28.16	35.24	35.84	37.50	19.24	21.48	26.68
Meilong Town	9.11	15.95	19.58	16.34	19.04	8.33	11.14	14.21
Zhuanqiao Town	7.11	19.24	25.96	21.21	24.05	11.83	19.31	18.39
Maqiao Town	20.15	35.26	42.32	35.43	39.48	22.13	19.77	30.65
Wujing Town	14.79	32.08	29.09	23.15	20.11	13.39	14.22	20.98
Pujiang Town	11.71	14.20	9.28	7.62	16.13	5.89	7.89	10.39
Xinhong Street	19.89	34.85	40.70	41.59	40.33	21.85	33.87	33.30
Gumei Street	6.67	18.90	31.16	23.79	16.61	11.76	14.54	17.63
Pujin Street	6.12	16.95	8.03	6.11	12.64	10.88	16.46	11.03
Jiangchuan Street	14.67	35.53	42.94	39.89	44.26	23.89	33.01	33.46

Vaccination coverage rates from 2013–2014 to 2019–2020 in each town/street were calculated according to children’s residential addresses. The mean coverage rate is the average coverage rate of that town/street from 2013–2014 to 2019–2020. ^†^Gray shading indicates that the vaccination coverage rate is below the mean in that column. ^††^Underscoring indicates that the vaccination coverage rate is below the lower quartile in that column.

Due to insufficient surveillance data compared to vaccination data, the number of influenza cases among registered children between 2013–2014 and 2017–2018 was estimated using incidence data and vaccination data (Supplementary Table S2). As newborn children continued to join the study cohort, the population of children and estimated number of influenza cases both increased substantially, from 24,652 and 4.27 in 2013–2014 to 126,054 and 77.41 in 2017–2018, respectively (Table 1 and Supplementary Table S2). Influenza vaccination prevented an average of 5.52 (range: 4.46–6.65) cases per year during the 5 years (Table 3). The largest number of averted cases occurred during 2017–2018, when 15.34 (range: 12.38–18.51) influenza cases were prevented by vaccination, corresponding to a prevented fraction of 16.54% (range: 13.79%-19.30). The year with the lowest number of averted events was 2013–2014, when 0.28 (0.23–0.34) cases were averted, with a prevented fraction of 6.15% (5.10–7.4%). The largest prevented fraction was 16.58% (range: 13.82–1.32%) in 2015–2016 when the vaccination coverage rate was the highest. Sensitivity analysis was also performed to test the influence of vaccination coverage on averted cases and prevented fractions (Supplementary Table S3). When vaccination coverage increased by 10%, the mean averted cases per year increased from 5.52 (range: 4.46–6.65) to 8.22 (range: 6.54–10.05), with an average improvement of 6% in the prevented fraction.

**Table 3:** Predicted impact of vaccination among children from 2013–2014 to 2017–2018.

	2013–2014	2014–2015	2015–2016	2016–2017	2017–2018
Vaccination coverage	10.40%	22.94%	27.62%	24.16%	27.57%
Estimated number of observed influenza cases	4.27	10.13	26.51	29.99	77.41
Estimated number of averted influenza cases (+/− 10%VE) ^†^	0.28 (0.23–0.34)	1.62 (1.31–1.94)	5.27 (4.25–6.35)	5.08 (4.12–6.10)	15.34 (12.38–18.51)
Prevented fraction (+/−10%VE)^††^	6.15% (5.11–7.38%)	13.79% (11.45–16.07%)	16.58% (13.82–19.32%)	14.49% (12.08–16.90%)	16.54% (13.79–19.30%)

The number of averted influenza cases among children was estimated using three parameters: number of observed influenza cases, vaccination coverage, and vaccine effectiveness. The number of observed cases was estimated using incidence data in Shanghai and the number of children in vaccination records. Vaccine effectiveness was assumed to be 60%. The results of sensitivity analyses with an interval of +/− 10% VE were presented as ranges of uncertainties. ^†^Number of averted influenza cases (NAC), computed as $\text{NAC}=\frac{n\cdot \text{VC}\cdot \text{VE}}{1-\text{VC}\cdot \text{VE}}$, where n: observed influenza cases, VC, vaccination coverage; VE, vaccine effectiveness. ^††^Prevented fraction (PF), calculated as $\text{PF}=\frac{\text{NAC}}{n+\text{NAC}}$, where NAC, number of averted influenza cases, n, observed influenza cases.

The impact of improving vaccination coverage in each town/street was also estimated by the ratio of the difference in the number of averted cases when increasing the vaccination coverage rate by 10% to observed cases (Table 4). The ratio was significantly positively correlated with the original vaccination coverage rate (*r* = 0.99, *p* < 0.001). The mean ratio in each area ranged from 7.27% on Pujin Street to 10.69% on Jiangchuan Street. Using a regression model, an original vaccination coverage rate of 31.53% was predicted to obtain a ratio of 10% when coverage was increased.

**Table 4:** The ratio of the difference in NAC to observed cases with a 10% increase in vaccination coverage.

Town/Street	2013–2014 (%)	2014–2015 (%)	2015–2016 (%)	2016–2017 (%)	2017–2018 (%)
Huacao Town	7.32	8.68	10.90	10.09	12.11
Qibao Town	6.91	8.40	8.72	8.18	9.29
Hongqiao Town	7.70	8.6	9.65	9.26	9.26
Xinzhuang Town	7.19	9.36	10.44	10.54	10.83
Meilong Town	7.16	7.86	8.26	7.90	8.20
Zhuanqiao Town	7.00	8.22	9.06	8.46	8.81
Maqiao Town	8.32	10.44	11.72	10.47	11.18
Wujing Town	7.75	9.94	9.49	8.70	8.33
Pujiang Town	7.42	7.67	7.19	7.03	7.88
Xinhong Street	8.28	10.38	11.41	11.58	11.34
Gumei Street	6.95	8.18	9.80	8.78	7.93
Pujin Street	6.90	7.97	7.07	6.89	7.51
Jiangchuan Street	7.72	10.49	11.85	11.26	12.11

NAC represents the number of averted cases, computed as $\text{NAC}=\frac{n\cdot \text{VC}\cdot \text{VE}}{1-\text{VC}\cdot \text{VE}}$. The difference in NAC was calculated using the formula when increasing the VC by 10%. The observed cases were distributed according to the proportion of qualified children in each town/street. The ratio was calculated as the difference in NAC divided by the observed cases.

**Figure 1:**
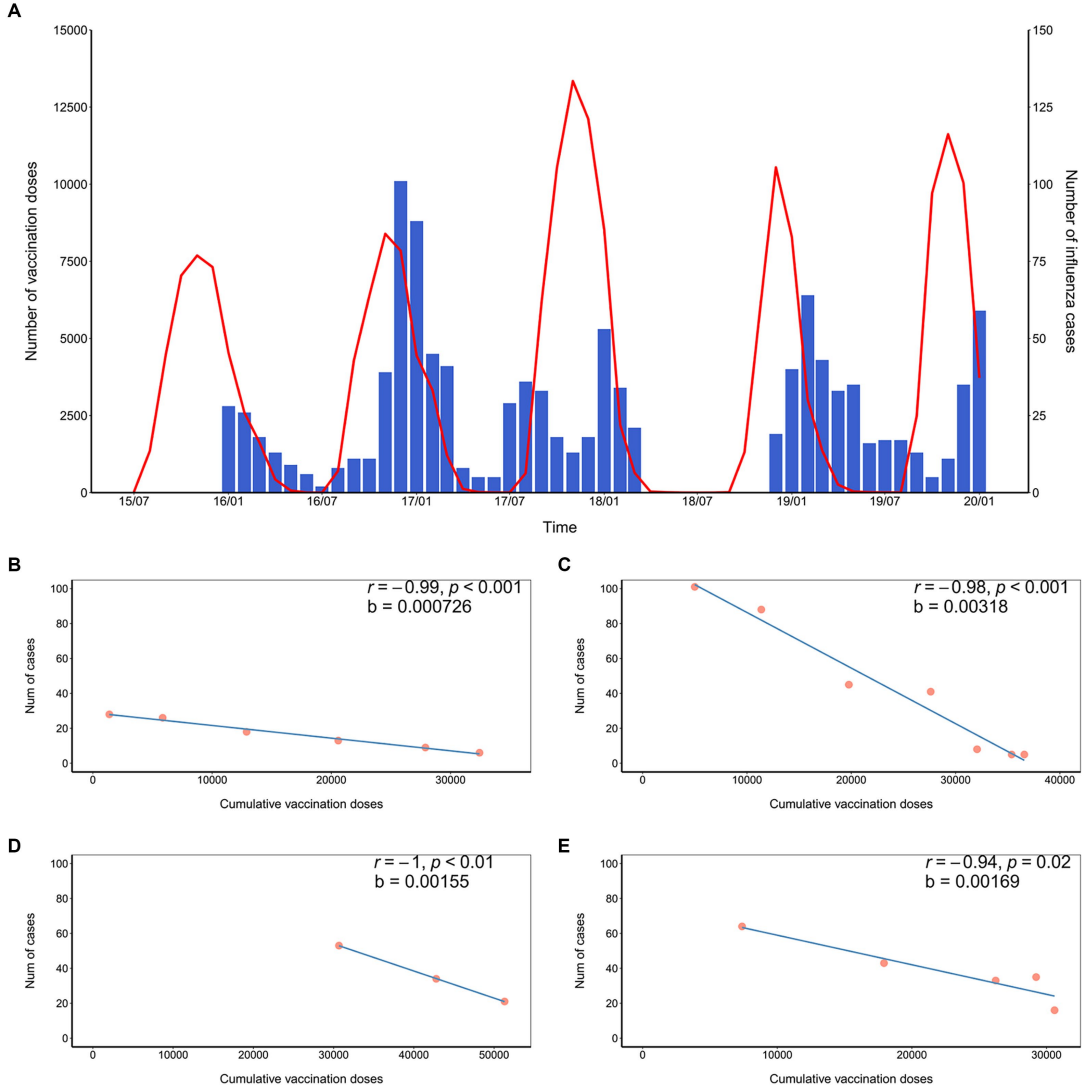
Monthly vaccination doses and influenza cases from July 2015 to January 2020 and the fitted regression line. **(A)** Monthly number of vaccination doses and influenza cases from July 2015 to January 2020. The left *y*-axis represents the number of vaccination doses per month. The right y-axis represents the number of reported influenza cases per month. The vaccination doses among children are shown by a red line. The number of notified influenza cases is shown by a blue bar, with data unavailable before January 2016 and data missing from April to November 2018; **(B)** Influenza cases from January to June 2016 fitted with cumulative vaccination doses from August 2015 to January 2016; **(C)** Influenza cases from December 2016 to June 2017 fitted with cumulative vaccination doses from September 2016 to March 2017; **(D)** Influenza cases from January to March 2018 fitted with cumulative vaccination doses from November 2017 to January 2018; **(E)** Influenza cases from February to June 2019 fitted with cumulative vaccination doses from December 2018 to March 2019.

Confirmed influenza cases in the general population in Minhang District from January 2016 to January 2020 with missing data from April to November 2018 are presented in Figure 1. The peak of influenza mainly arose in the winter months, with an exception in the summer of 2017. To quantify the relationship between the decrease in influenza cases in all age groups and the cumulative vaccination doses in prior or identical months, four time periods when the case counts declined were selected: January–June 2016, December 2016–June 2017, January–March 2018, and February–June 2019. The most correlated results between the number of influenza cases in the selected periods and the cumulative vaccination volume in that vaccination year were summarized (Table 5). The number of influenza cases from January to June 2016 was most strongly correlated with cumulative vaccination doses 5 months before, with a correlation coefficient of −0.99. There was a time lag of 3 months between cases in both of the periods, December 2016 to June 2017 and February to June 2019, and the most correlated cumulative vaccination doses, of which correlation coefficients were − 0.98 and − 0.94, respectively. The number of cases from January to March 2018 was completely negatively correlated with cumulative vaccinations 2 months before. Regressions on the influenza cases and cumulative vaccination doses in most correlated months were conducted to estimate the reduction in influenza cases per thousand additional vaccinations (Table 5). An estimated reduction of 3.18 cases occurred from December 2016 to June 2017 with 1,000 additional vaccinations from September 2016 to March 2017, which was the greatest reduction estimated. The smallest reduction was 0.73 cases from January to June 2016, with 1,000 additional vaccinations from August 2015 to January 2016. The estimated case reductions climbed upward in the first 2 years and then fell to a moderate level in the following 2 years, which equaled 1.55 and 1.69 per 1,000 additional vaccinations, respectively.

**Table 5:** Relationship between cumulative vaccination doses and influenza cases.

Surveillance periods	Vaccination periods	r ^†^	pvalue	b ^††^
16/01–16/06	15/08–16/01	−0.99	<0.001	$-7.26\times 10^{-4}$
16/12–17/06	16/09–17/03	−0.98	<0.001	$-3.18\times 10^{-3}$
18/01–18/03	17/11–18/01	−1.00	0.01	$-1.55\times 10^{-3}$
19/02–19/06	18/11–19/03	−0.94	0.02	$-1.69\times 10^{-3}$

Four surveillance periods in which the number of influenza cases decreased were selected. Correlation analyses were conducted between the number of influenza cases in surveillance periods and the number of cumulative vaccination doses in prior or identical months in the same vaccination year. Only the most correlated results for each period are listed here. Then, the reduction in influenza cases per additional vaccination dose was estimated by the regression coefficient between the number of influenza cases and cumulative vaccination doses. ^†^*r*, Pearson’s correlation coefficient. ^††^b, regression coefficient, represents the estimated reduction in influenza cases per additional vaccination dose.

## 4. Discussion

The aim of this study was to explore whether influenza vaccination among children could provide direct effects in themselves and indirect effects in the general population by using surveillance data of the vaccination records for children born between 2013 and 2019, and of the reported influenza cases between 2016 and 2020.

There were several findings arising from the present study. First, the influenza vaccination rate was suboptimal among children in Minhang considering the goal of 75% coverage proposed by the WHO recommendation (31). Second, the vaccination program could provide direct protections to children as an average fraction of 13.15% of potential influenza cases was estimated to be averted. Third, the indirect effects provided by inoculating children were observed as a strong negative correlation between the cumulative number of influenza vaccinations and the number of cases with time lags. Finally, improving the vaccination coverage rates in higher coverage areas were estimated to be associated with more averted cases.

In this study, the annual influenza vaccination coverage among children in the vaccination records ranged from 10.40 to 27.62%, with a mean coverage of 20.71%. This vaccination coverage of influenza in Minhang was lower than that reported in other studies. A study revealed that the coverage was 59.15% among children at high risk of influenza in the United States (32), and similarly, a coverage of 58.28% was documented in the United Kingdom according to WHO Data Portal (33). In China, a meta-analysis found that the pooled influenza vaccination coverage was 28.4% for children aged 6 months to 5 years (13). There may be several reasons that contributed to the low coverage rate. First, the supply of influenza vaccines available to young children in the study period was insufficient. Second, the vaccination procedure is cumbersome. Regardless of the history of influenza vaccination, children need to be vaccinated before the influenza season each year. Otherwise, children aged 6 months to 3 years need to receive two doses of influenza vaccine with an interval of more than 4 weeks (34). In addition, parents tend to regard influenza vaccines as being of little importance and refuse to pay out-of-pocket (35), as they are not included in the national immunization program in China. Consequently, parents do not prioritize immunizing their children with influenza vaccines in the absence of an influenza epidemic.

Despite the comparatively low vaccination coverage, this study identified the direct protective effects of inoculating children by an average estimation of 13.51% of averted cases. The impact of influenza vaccination programs varied across the influenza seasons, ranging from 6.15 to 16.58%. This result is higher compared to a study conducted in Suzhou, China, that reported an average prevented fraction of 7% over five seasons (17). The difference may attribute to higher vaccination coverage rate of 21% in this study than 9% in the latter. Similarly, a research reported an average prevented fraction of 53% when coverage rate reaching 46% (19). However, the number of averted cases was estimated to be low, even close to zero in 2013–2014. This reflected the low number of influenza cases that year and that few cases can be prevented when the underlying incidence is low (21). Since the number of influenza cases were based on the statistics of influenza incidence from the China CDC, it may underestimate the real burden. As the sample submitted for confirmed diagnosis and influenza subtyping is usually a nasopharyngeal swab or serum specimen, and collecting these samples is an invasive, sometimes painful experience, children and even parents are reluctant to cooperate. In addition, infected children with mild symptoms generally choose to receive treatment in clinics or community health centers that are not included in the infectious disease reporting system. Therefore, reported childhood cases are inpatients with serious symptoms to a large extent. As a result, the estimations in this study can be regarded as the number of severe cases.

Due to the lack of reliable evidence of VE in Shanghai and the wide variations in VE, simulated vaccine effectiveness was used in this study. A meta-analysis study reported pooled vaccine effectiveness against types of influenza ranging from 43 to 69% in pediatric age groups (28). To assess the benefit of vaccination, the influenza vaccine was assumed to have a moderate effectiveness of 60% in our study, and the results of the sensitivity analyses of VE were presented as ranges of estimates to indicate uncertainties. Compared to the upper limit of the uncertainty range, the increment in vaccination impact estimates was larger when improving VC by 10%. As VE varies across seasons, populations, age groups, and products (36), it is more beneficial to focus on measures aiming to improve vaccination coverage.

This study also measured the influence of increasing the vaccination coverage rate in each town/street by the ratio of the difference in NAC to observed influenza cases. The ratio represented what fraction of cases could be further averted on the basis of observed cases when increasing the vaccination coverage rate by 10%. The observed cases were proportional to the number of children who met the criteria for vaccination in each area. The results indicated that a larger proportion of influenza cases can be averted in areas of higher coverage than in lower coverage areas when increasing the coverage rate. Thus, areas with high vaccination coverage should further increase the rates to avoid more influenza cases and even establish herd immunity. In this simulation, Jiangchuan, Maqiao and Xinhong received more benefits than other areas from the increase in coverage. We also calculated a threshold of 31.53% of the original vaccination coverage to achieve a 10% ratio.

This study quantified the relationship between influenza cases in the general population and cumulative vaccination doses among children from two aspects. First, a strong negative correlation was observed, which implied underlying indirect protection provided by vaccinating children. This is consistent with previous studies, in which statistically significant indirect protection by inoculating children was found ranging from 24 to 61% (11, 37, 38). The time lag between cumulative vaccination doses and influenza cases ranged from 2 to 5 months, with a median of 3 months. The two periods with complete surveillance data were found in a three-month gap, while the remaining periods with incomplete surveillance data had a time lag of two and 5 months. Incomplete data may have contributed to the variations in time lags. Second, we estimated the reduction in influenza cases in the general population per thousand additional vaccinations among children. The estimated reduction increased from 0.73 cases to 3.18 cases in the first two periods and then fell to a moderate level of 1.55 and 1.69 cases. The mismatch between the vaccine strain and circulating viruses may have resulted in the small estimated reduction in influenza cases from January to June 2016 (39, 40).

This study described the trend in influenza vaccination coverage among children from a spatiotemporal perspective for 7 consecutive years in Minhang District. The method to estimate averted cases used in this study has been widely used to evaluate the impact of influenza vaccination (18, 21, 22, 25). However, this is the first study to apply this method to estimate the impact of influenza vaccination among children in China. Compared to other estimation models, this method involved fewer intensive computations and easily interpretable results. The relationship between influenza cases and cumulative vaccination doses was quantified to explore the underlying benefit provided by the vaccination of children.

There were some limitations in this study. First, we could not stratify children’s age to calculate age-specific influenza vaccination coverage rates. As newborn infants were enrolled in this cohort successively, the coverage rate was computed by the number of vaccinated children divided by the total number of children who were eligible for vaccination in one vaccination year. Second, the number of influenza cases among children from 2013 to 2018 was estimated using influenza incidence data in Shanghai multiplied by the number of children in vaccination records rather than the surveillance data. The difference in influenza incidence among children between Shanghai and Minhang may have introduced an underestimation or overestimation in later estimations. Third, the method used did not consider the indirect effects of the vaccination program and thus presented a more conservative estimate of the impact. Moreover, the annual averted cases could only be estimated within limited age groups, as we used the vaccination data of a birth cohort. Finally, we did not have VE data specifically for Minhang District for the study period, and the simulated level may not reflect the real effectiveness of the influenza vaccine.

The current study suggests that influenza vaccinations among children could offer both direct and indirect protections, which emphasize the importance of increasing influenza vaccination coverage to reduce influenza morbidity. These results provide easily interpretable evidence for childhood vaccination to public health recommendations and can be particularly useful in countries including China currently developing influenza vaccination policies.

## 5. Conclusion

In summary, the current study identified the distribution of influenza vaccination coverage in Minhang District on a spatiotemporal scale. Vaccination coverage in high-coverage areas should be further expanded to avert more influenza cases and even to establish herd immunity. In low-coverage areas, a threshold of 31.53% was estimated to maximize the benefit of vaccinations. Vaccinations among children averted an average of 13.51% of influenza cases per year. The results of quantifying the relationship between cumulative vaccination doses and influenza cases indicated that the influenza vaccine program among children was strongly correlated with influenza activities and might provide underlying indirect protection to the general population.

## Data availability statement

The data analyzed in this study is subject to the following licenses/restrictions: the dataset is part of Minhang CDC Notifiable Communicable Disease Reporting System and the National Immunization Program System, it’s not public available according to the legislations. Requests to access these datasets should be directed to yyao@fudan.edu.cn.

## Ethics statement

The studies involving humans were approved by the Institutional Review Board of the Minhang Center for Disease Control and Prevention. The studies were conducted in accordance with the local legislation and institutional requirements. The ethics committee/institutional review board waived the requirement of written informed consent for participation from the participants or the participants’ legal guardians/next of kin because this study involved the use of surveillance data and no potentially identifiable human data were presented.

## Author contributions

YY and YL contributed to the conception and design of the study. NL, BJ, and KM organized the database. LS performed the statistical analysis and wrote the first draft of the manuscript. ZZ, LZ, and XZ contributed to the discussion and revision of the manuscript. JL provided the financial support for the manuscript. All authors contributed to manuscript revision, read, and approved the submitted version.

## Funding

This study was supported by the Talent Program by Minhang District Health Commission (2020FM29), Minhang Public Health Brand Department (MGWKS01), the Shanghai Municipal Health Commission Clinical Research Program (20214Y0020), the General Program of Natural Science Foundation of Shanghai Municipality (22ZR1414600), and the Young Health Talents Program of Shanghai Municipality (2022YQ076).

## Conflict of interest

The authors declare that the research was conducted in the absence of any commercial or financial relationships that could be construed as a potential conflict of interest.

## Publisher’s note

All claims expressed in this article are solely those of the authors and do not necessarily represent those of their affiliated organizations, or those of the publisher, the editors and the reviewers. Any product that may be evaluated in this article, or claim that may be made by its manufacturer, is not guaranteed or endorsed by the publisher.
