# Supplementary material for: Coverage and impact of influenza vaccination among children in Minhang District, China, 2013–2020
Source: Front Public Health. 2023 Aug 30;11:1193839. doi: 10.3389/fpubh.2023.1193839 (PMC10499390; doi:10.3389/fpubh.2023.1193839)
Supplement: Supplementary file 1 [file Table_1.DOCX]

Supplementary Material

Coverage and Impact of Influenza Vaccination among Children in Minhang District, China, 2013-2020

**Zhaowen Zhang^†^, Liming Shi^†^, Nian Liu, Biyun Jia, Kewen Mei, Liping Zhang,**

**XuanZhao Zhang, Yihan Lu, Jia Lu* and Ye Yao***

*** Correspondence:** Jia Lu: cpulj@126.com, Ye Yao: yyao@fudan.edu.cn

Supplementary Table 1 The demographic characteristics of vaccinated and unvaccinated children during 2013-2020

|  | Vaccinated  (n = 78027) | Unvaccinated  (n = 92888) | $\chi^{2}$ | *P* value |
| --- | --- | --- | --- | --- |
| Sex |  |  |  |  |
| Male | 40917 | 48496 | 0.90 | 0.34 |
| Female | 37110 | 44392 |  |  |
| Residency |  |  |  |  |
| Permanent | 40607 | 44963 | 224.33 | <0.001 |
| Nonpermanent | 37420 | 47925 |  |  |
| Address |  |  |  |  |
| Huacao Town | 6364 | 4106 | 6500 | <0.001 |
| Qibao Town | 8429 | 10015 |  |  |
| Hongqiao Town | 5197 | 5673 |  |  |
| Xinzhuang Town | 12197 | 9744 |  |  |
| Meilong Town | 6377 | 11017 |  |  |
| Zhuanqiao Town | 8372 | 9875 |  |  |
| Maqiao Town | 4895 | 4099 |  |  |
| Wujing Town | 5108 | 5433 |  |  |
| Pujiang Town | 5362 | 13642 |  |  |
| Xinhong Street | 2057 | 1622 |  |  |
| Gumei Street | 4436 | 6390 |  |  |
| Pujin Street | 2765 | 6043 |  |  |
| Jiangchuan Street | 6468 | 5229 |  |  |

All children included in this study were categorized as the vaccinated group if they were vaccinated at least 1 dose of influenza vaccine during the study period, otherwise as the unvaccinated group. A chi-square test was conducted to analyze the demographic features between the two groups.

Supplementary Table 1 The demographic characteristics of vaccinated and unvaccinated children during 2013-2020 (Continued)

Supplementary Table 2 The estimated influenza cases from 2013-2014 to 2017-2018

|  | 2013-2014 | 2014-2015 | 2015-2016 | 2016-2017 | 2017-2018 |
| --- | --- | --- | --- | --- | --- |
| Vaccine coverage rate (%) | 10.40 | 22.94 | 27.62 | 24.16 | 27.57 |
| Estimated influenza cases | 4.27 | 10.13 | 26.51 | 29.99 | 77.41 |

The number of influenza cases was estimated by multiplying the monthly count of children in each age group in the vaccination records by the monthly influenza incidence rate in Shanghai and then adding the monthly estimations together in each age group to get a yearly count.

Supplementary Table 3 Predicted impact of vaccinations among children with an increase of 10% in vaccination coverage, from 2013-2014 to 2017-2018

|  | 2013-2014 | 2014-2015 | 2015-2016 | 2016-2017 | 2017-2018 |
| --- | --- | --- | --- | --- | --- |
| Vaccine coverage | 20.40% | 32.94% | 37.62% | 34.16% | 37.57% |
| Estimated number of observed influenza cases | 4.27 | 10.13 | 26.51 | 29.99 | 77.41 |
| Estimated number of averted influenza cases (+/- 10%VE) ^†^ | 0.59  (0.48-0.71) | 2.50  (2.00-3.04) | 7.73  (6.14-9.48) | 7.73  (6.18-9.42) | 22.53  (17.90-27.62) |
| Prevented fraction  (+/-10%VE) ^††^ | 12.14%  (10.11%-14.26%) | 19.79%  (16.49%-23.08%) | 22.58%  (18.81%-26.34%) | 20.49%  (17.09%-23.90%) | 22.54%  (18.78%-26.30%) |

The number of averted influenza cases among children was estimated using three parameters: the number of observed influenza cases, vaccination coverage, and vaccine effectiveness. The number of observed cases was estimated using incidence data in Shanghai and the number of children in vaccination records. Vaccine effectiveness was assumed to be 60%. Results of sensitivity analyses with an interval of +/- 10% VE were presented as ranges of uncertainties.

^†^ Number of averted influenza cases (NAC), computed as $NAC=\frac{n\cdot VC\cdot VE}{1-VC\cdot VE}$, where n: observed

influenza cases, VC: vaccination coverage, VE: vaccine effectiveness.

^††^ Prevented fraction (PF), calculated as $PF = NAC/(n+NAC),$ where NAC: number of averted influenza cases, n: observed influenza cases.


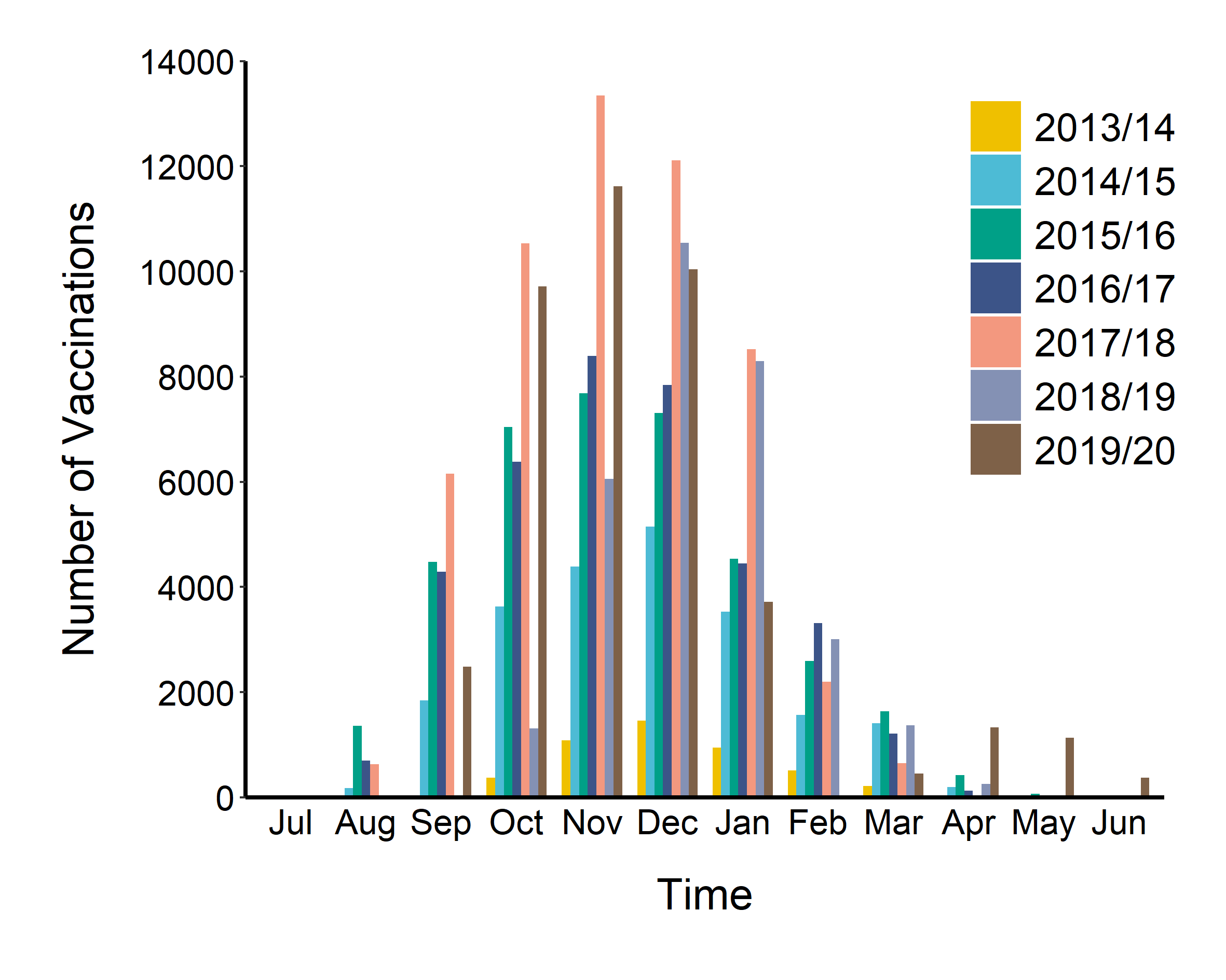


**Supplementary Figure 1.** **Monthly vaccination doses among children in Minhang District from 2013-2014 to 2019-2020.** Each column represents the number of vaccination doses among children in that month.
